# The *PIK3CA* gene and its pivotal role in tumor tropism of triple-negative breast cancer

**DOI:** 10.1016/j.tranon.2024.102140

**Published:** 2024-10-05

**Authors:** Sumit Mallick, Asim K Duttaroy, Suman Dutta

**Affiliations:** a Stem Cells and Regenerative Medicine Centre, Yenepoya Research Centre, Yenepoya (Deemed to be University), University Road, Mangalore, Karnataka, India; b Department of Nutrition, Faculty of Medicine, Institute of Basic Medical Sciences, University of Oslo, Norway; c Nuffield Department of Clinical Neurosciences, John Radcliffe Hospital, University of Oxford, Oxford, UK; d Dorothy Crowfoot Hodgkin Building, Kavli Institute for Nanoscience Discovery, University of Oxford, Oxford, UK

**Keywords:** Triple negative breast cancer, Tumor tropism, PI3K, EMT, PI3K/AKT/mTOR pathway, Metastasis, Targeted therapy

## Abstract

The *PIK3CA* gene is a linchpin in the intricate molecular network governing triple-negative breast cancer (TNBC) tumor tropism, serving as a focal point for understanding this aggressive disease. Anchored within the PI3K/AKT/mTOR signaling axis, *PIK3CA* mutations exert substantial influence, driving cellular processes that highlight the unique biology of TNBC. This review meticulously highlights the association between *PIK3CA* mutations and distinct TNBC subtypes, elucidating the gene's multifaceted contributions to tumor tropism. Molecular dissection reveals how *PIK3CA* mutations dynamically modulate chemokine responses, growth factor signaling, and extracellular matrix interactions, orchestrating the complex migratory behaviour characteristic of TNBC cells. A detailed exploration of *PIK3CA*-targeted strategies in the therapeutic arena is presented, outlining the current landscape of clinical trials and precision medicine approaches. As the scientific narrative converges, this review underscores the critical role of *PIK3CA* in shaping the molecular intricacies of TNBC tumor tropism and illuminates pathways toward tailored interventions, promising a paradigm shift in the clinical management of TNBC.

## Highlights

• *PIK3CA*, the most frequently mutated gene in breast cancer, influences aggressive behaviour in TNBC and promotes tumor tropism.
• Mutation in the *PIK3CA* gene can affect the epithelial-mesenchymal transition (EMT), fostering a more invasive and migratory TNBC phenotype.
• Current clinical trials assess combinations of hormone therapy and dual inhibitors for targeting the *PIK3CA*-mutated breast cancer subtypes.

## Graphical abstract

**Figure:**
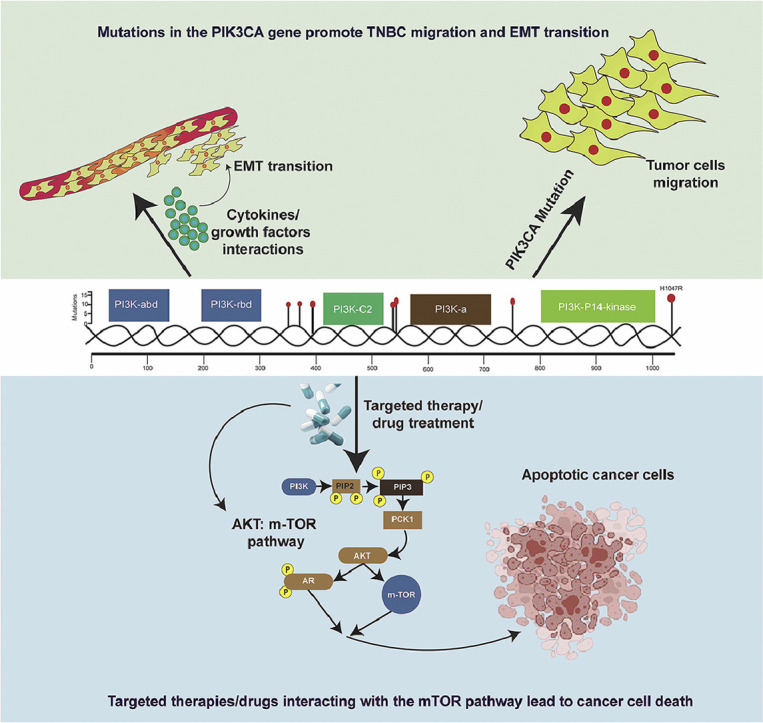

**Key message**

*PIK3CA* mutation is the second most common event in all types of breast cancer, with a higher rate in hormone receptor-positive breast cancer compared to TNBC. Alteration of the PI3K pathway and *PIK3CA* mutation plays a key role in tumor tropism and metastasis in breast cancer (BC). Despite promising results from recent clinical trials targeting *PIK3CA*-mutated tumors, challenges remain within the PI3K signaling pathway. These challenges include tumor heterogeneity, the development of resistance, and the need to address processes such as angiogenesis and cell survival. Ongoing research seeks to overcome these hurdles, refining *PIK3CA*-targeted therapies and developing alternative or combination approaches [[1], [2], [3]].

Alt-text: Unlabelled box

## Introduction

TNBC represents a significant clinical challenge in the landscape of breast cancer due to its unique molecular profile characterized by the absence of estrogen receptor (ER), progesterone receptor (PR), and human epidermal growth factor receptor 2 (HER2) expression [4,5]. This "triple-negative" classification limits the efficacy of targeted therapies commonly used in other breast cancer subtypes, necessitating reliance on conventional chemotherapy [4]. TNBC exhibits an inherently aggressive nature, marked by higher histological grade, increased proliferative activity, and an elevated propensity for metastasis [6]. Its aggressive behaviour contributes to a poorer prognosis and limited treatment options, making TNBC a formidable adversary in breast cancer [7].

Challenges in treating TNBC are multifaceted. The lack of well-defined molecular receptors such as ER, PR, and HER2 renders hormone therapies and HER2-targeted agents ineffective, leaving chemotherapy as the primary systemic treatment option. However, the non-specific nature of chemotherapy also contributes to increased toxicity, highlighting the urgent need for targeted therapeutic strategies. A central challenge in managing TNBC is its molecular heterogeneity, encompassing distinct subtypes with diverse clinical behaviours and responses to treatment. The presence of stem cell populations within tumors further contributes to this heterogeneity and enhances aggressiveness [[8], [9], [10]]. This inherent heterogeneity not only affects the clinical trajectory of the disease but also present challenges in devising standardized therapeutic strategies.

For instance, tumor tropism — the tendency of cancer cells to migrate and colonize specific distant sites —is particularly relevant to TNBC. Understanding tumor tropism is crucial for elucidating the mechanisms underlying metastasis, which is often associated with a poor prognosis in TNBC patients [11]. Furthermore, TNBC's unique biology underscores the need for a deeper understanding of the molecular mechanisms driving its aggressive phenotype, especially in relation to tumor tropism. Tumor tropism involving the selective migration and homing of cancer cells to specific tissues or organs, adds a layer of complexity to TNBC [12]. Deconstructing the molecular intricacies of tumor tropism is imperative for unveiling novel therapeutic avenues and addressing the challenges posed by the aggressive nature of TNBC [13].

**Fig. 1:**
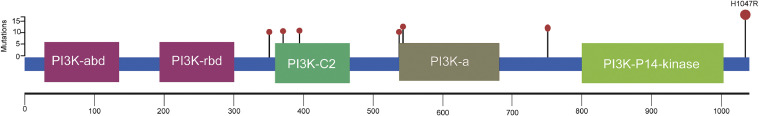
Mutational spectrum of *PIK3CA* in TNBC subtypes. This figure illustrates the protein domains of PIK3CA and highlights the positions of specific mutations commonly found in TNBC subtypes. The most frequent mutation type in each domain is labeled, with red dots representing missense mutations.

In this review, we focus on the *PIK3CA* gene and its role in tumor tropism. The *PIK3CA* gene, encoding a key protein in the PI3K signaling pathway, is mutated in about 40% of TNBC cases and also frequently occurs in hormone receptor-positive (HR+) disease [14]. This *PIK3CA*-associated PI3K pathway regulates cell migration and invasion. Recent studies have explored the association between *PIK3CA* mutations (Fig. 1) and TNBC tropism. PI3Ks have a critical role in the control of various cellular processes such as cell growth and proliferation, metabolism, and migration via the PI3K/AKT/mTOR pathway. The *PIK3CA* gene also plays a crucial in tumor metastasis and tropism [15]. Therefore, elucidating the molecular intricacies of tumor tropism is crucial for developing novel therapeutic strategies, yet much remains unknown about its specific drivers in TNBC. Against this backdrop, the *PIK3CA* gene emerges as a key player in TNBC's aggressive phenotype, potentially influencing tumor tropism through the modulation of complex signaling pathways. Furthermore, we explore the intricate signaling pathways modulated by PIK3CA, with a particular focus on their influence on tumor tropism in breast cancer, emphasizing TNBC.

## The role of the *PIK3CA* gene in intracellular signaling pathways under physiological and TNBC conditions

**Fig. 2:**
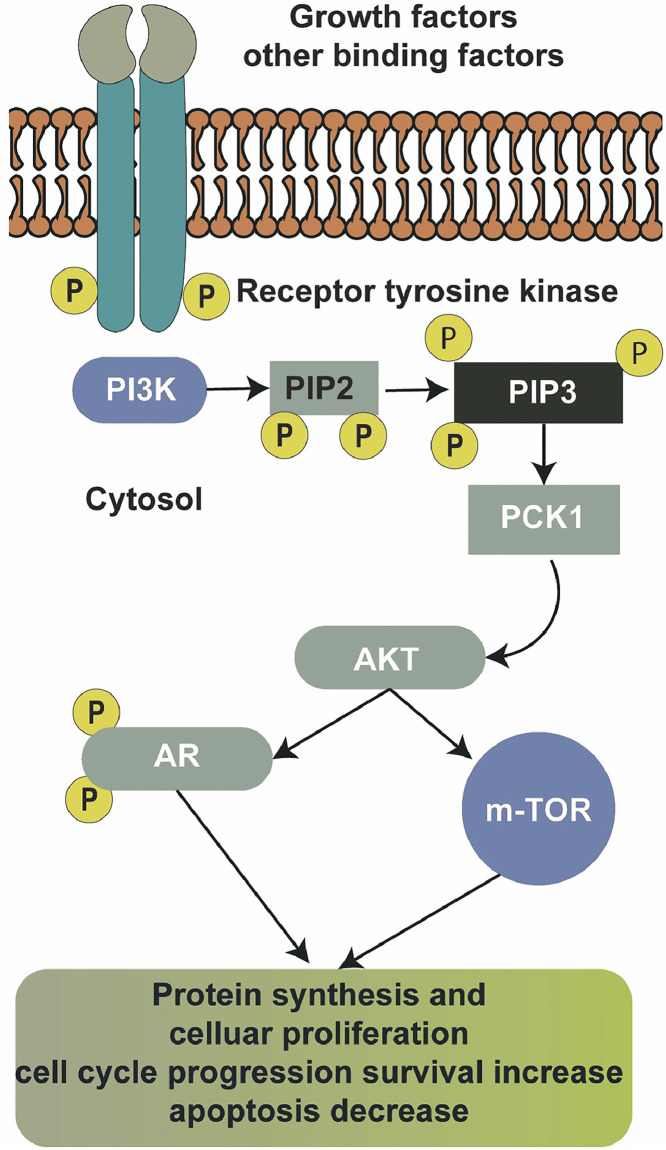
PI3K and PIP2-mediated pathway. This figure depicts the PI3K-mediated phosphorylation of PIP2 to PIP3, which activates downstream effectors, including AKT. These effectors are crucial for promoting cell survival, growth, and migration in TNBC cells.

The *PIK3CA* gene is a key player in intracellular signaling pathways under both physiological and TNBC conditions. Understanding the association between *PIK3CA* mutations and specific TNBC subtypes provides valuable insights for personalized treatment strategies (Fig. 1). The *PIK3CA* gene on chromosome *3q26.3* encodes the catalytic subunit of PI3K, a key enzyme in the PI3K/AKT/mTOR signaling pathway [16]. The gene spans approximately 34 kg bases and consists of 20 exons. *PIK3CA*'s structure encompasses domains critical to its function, including the p85-binding, helical, and kinase domains [17]. The PI3K/AKT/mTOR pathway, regulated by *PIK3CA*, plays a pivotal role in cellular processes governing proliferation, survival, and metabolism [18]. A series of downstream molecules is activated when growth factors such as EGFR and VEGFR bind to their receptors. Next, PI3K, which is present in the cytosol, converts phosphatidylinositol 4,5-bisphosphate (PIP2) to phosphatidylinositol (3,4,5)-trisphosphate (PIP3) (Fig. 2) [19,20]. PIP3 recruits AKT to the cell membrane, facilitating its activation by phosphorylation. Activated AKT subsequently phosphorylates downstream effectors, including mTOR, orchestrating a cascade of events promoting protein synthesis and cell growth [21]. Thus, the *PIK3CA* gene is pivotal in intracellular signaling pathways, particularly in TNBC. Anchored in the PI3K/AKT/mTOR signaling axis, *PIK3CA* encodes the catalytic subunit of PI3K and plays a major role in cellular growth and metastasis [22]. Aberrations in the *PIK3CA* gene, often in the form of activating mutations, have emerged as recurrent events in various cancer types, including TNBC. In TNBC, *PIK3CA* mutations contribute significantly to the molecular landscape, fuelling the dysregulation of intracellular signaling cascades. Activation of the PI3K/AKT/mTOR pathway, driven by *PIK3CA* mutations, can confer a survival advantage to cancer cells, promoting uncontrolled proliferation and resistance to apoptosis [19].

The potential relevance of *PIK3CA* to TNBC extends beyond its role in sustaining proliferative signals. It encompasses a broader impact on various aspects of TNBC biology and aggressiveness. Emerging evidence suggests that *PIK3CA* mutations may influence TNBC's aggressive behaviour, impacting invasiveness and metastatic potential, leading the TNBC cells to exhibit tropism [23]. Moreover, the intricate interplay between *PIK3CA* and the tumor microenvironment within TNBC remains a focal point of the investigation, revealing the gene's multifaceted role in shaping the complex biology of this breast cancer subtype. Understanding the significance of *PIK3CA* in TNBC is integral to unraveling the disease's molecular heterogeneity and devising targeted therapeutic strategies [24]. This intricate signaling cascade, tightly regulated by *PIK3CA*, ultimately influences various cellular processes, including those that govern a tumor cell's propensity for migration and invasion – hallmarks of tumor tropism.

## TNBC subtypes and their role in biological processes

In 2018, Bareche et al. identified six TNBC subtypes based on genomic profiling. They are categorized as BL-1, BL-2, IM, M, MSL, and LAR subtypes (Table 1) [25]. Moreover, Lehmann et al. (2021) have also explored the vulnerabilities of the TNBC subtypes by using a multi-omics approach [26]. BL1 tumors displayed high chromosomal instability, with mutations in TP53 (92%) and MUC16 (21%), alongside frequent mutations and amplifications as well as deletions in DNA repair genes Conversely, the LAR subtype harbored a higher mutational burden with enriched mutations in *PI3KCA* (55%), KMT2C (19%), NF-1 (13%), AKT1 (13%), and CDH1 (13%) [[25], [26], [27]]. Mesenchymal and MSL subtypes exhibited elevated angiogenesis signatures. As expected, the IM group expressed high levels of immune response genes and checkpoint inhibitors (CTLA-4, PD-1, PD-L1), potentially due to immune cell infiltration [28]. Interestingly, the LAR subtype is associated with a poorer prognosis, while the IM subtype is associated with a more favorable outcome [28]. Furthermore, Bareche et al. (2018) confirmed the reproducibility of BL1, IM, LAR, M, and MSL subtypes, aligning with previous observations of Lehmann's classification and limitations regarding BL2 and UNS subtypes [25].

**Table 1:** TNBC subtypes and their role in various biological pathways: This table categorizes TNBC into four subtypes: Basal-like 1 (BL-1), Basal-like 2 (BL-2), Immunomodulatory (IM), Mesenchymal (M), Mesenchymal Stem-like (MSL), and Luminal Androgen Receptor (LAR). Each subtype is associated with specific biological pathways, highlighting their roles in the diverse molecular mechanisms driving TNBC progression and response to therapy.

TNBC subtypes	Involved in biological functions	Interactive pathways
BL-1 & BL-2	**BL-1:** Cell cycle and cell division components (cell cycle, DNA replication reactome, G2 cell-cycle pathway, RNA polymerase, and G1 to S cell cycle). **BL-2:** Growth factor signaling, glycolysis, and gluconeogenesis.	The BL2 subtype is involved in a few gene ontologies like growth factor signaling • EGF pathway • NGF pathway • MET pathway • Wnt/β-catenin pathway • IGF1R pathway.
IM subtype	This subtype is especially involved in immune cell processes.	• Immune cell signaling (TH1/TH2 pathway, • NK cell pathway, • B cell receptor [BCR] signaling pathway, • DC pathway and T cell receptor signaling pathway • Cytokine signaling pathway, • IL-12 pathway • IL-7 pathway • Antigen processing and presentation, and signaling through core immune signal transduction pathways (NFKβ, TNF, and JAK/STAT signaling).
M and MSL subtypes	Involved in cell motility.	• ECM receptor interaction, and cell differentiation pathways • Wnt pathway • Anaplastic lymphoma kinase (ALK)pathway and • TGF-β signaling pathway
LAR subtype	Involved in androgen receptor signaling and showed luminal gene expression patterns [14].	• Androgens, estrogens, and porphyrins metabolism pathways • PARP signaling pathways.

## Association between *PIK3CA* mutations and TNBC subtypes

**Fig. 3:**
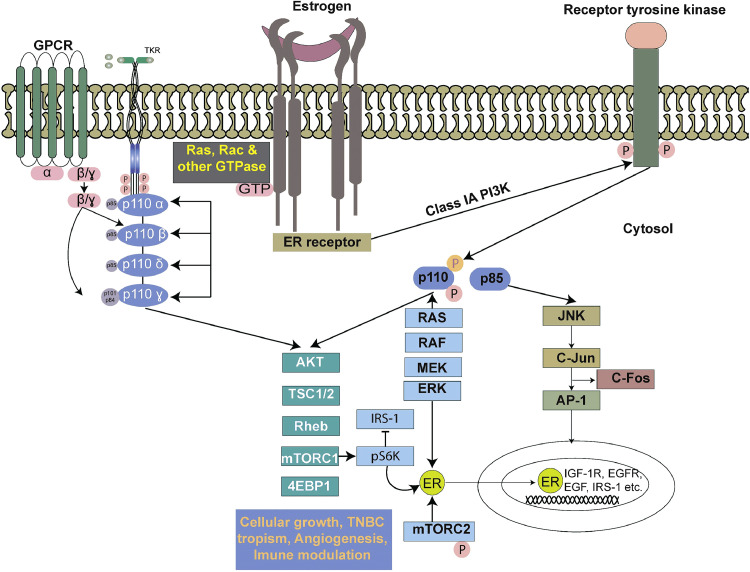
Overview of the PI3K/AKT/mTOR signaling pathway. This figure overviews the PI3K/AKT/mTOR signaling pathway, activated by estrogen and receptor tyrosine kinases. Key activating nodes (AKT, Rheb, mTORC1, and mTORC2) and negative regulators (PTEN, TSC complex) are highlighted. The figure also illustrates the crosstalk with the RAS and LKB1/AMPK pathways.

Understanding the association between *PIK3CA* mutations and specific TNBC subtypes provides valuable insights for personalized treatment strategies. PI3Ks are intracellular signal transducers that phosphorylate the 3‑hydroxyl group of phosphoinositide on the cell membrane. Among the various classes of PI3Ks, class I is the most frequently altered in breast cancer [29]. A PI3K is a heterodimer composed of a regulatory (p85) and catalytic subunit (p110). There are several isoforms of both regulatory subunits (p85α and p85β) and the catalytic subunits (p110α, p110β, p110γ, p110δ) (Fig. 3) [30].

*PIK3CA*, the gene encoding the p110α catalytic subunit of PI3K, was found to be frequently mutated in breast cancer (27%) [[29], [30], [31], [32]]. In TNBC, most activating mutations occur in the p110α subunit encoded by *PIK3CA*, with mutations present in 9% of primary TNBC [33]. However, the rate of mutations may increase in advanced TNBC, reflecting the high rate of observed *PIK3CA* mutations in initially ER^+^ breast cancer, which relapses, loses ER expression and becomes secondary TNBC while retaining the high rate of activating PI3Ks. *PIK3CA* mutations activate alpha PI3Ks in the cell membrane, increasing phosphorylation [34]. The association between *PIK3CA* mutations and TNBC subtypes is a pivotal aspect of molecular characterization, shedding light on the intricate heterogeneity within this aggressive breast cancer category [15,[35], [36], [37]]. Numerous studies, such as Kumar et al. (2021), and Lehmann et al. (2014), have discerned varying prevalence and distribution patterns of *PIK3CA* mutations across TNBC subtypes [[36], [37], [38], [39]]. Research conducted by Zardavas et al. (2018) highlighted that *PIK3CA* has a weaker association in the multivariate analysis than that found in univariate analysis, perhaps owing to the association of *PIK3CA* mutations with favorable clinical characteristics [40]. Specific subtypes exhibit higher frequencies of *PIK3CA* mutations, influencing clinical behaviours and treatment responses. This association underscores the imperative role of molecular profiling in stratifying TNBC patients based on their *PIK3CA* mutational status, providing a foundation for tailored therapeutic strategies. As elucidated by Liu et al. (2023), decoding the nuanced relationship between *PIK3CA* mutations and TNBC subtypes holds promise for advancing precision medicine, facilitating more targeted and productive interventions based on the unique genetic landscape of individual tumors [41]. Other studies have revealed that patients with TNBC are typically treated with DNA-based chemotherapy [42]. Mutations in PI3K pathways have been linked to resistance to these agents, likely by increasing cell survival. DNA damage triggers a response pathway involving DNA-dependent protein kinase (DNA-PK), which in turn phosphorylates AKT. Preclinical studies in various cancer cell lines have shown that inhibiting PI3K, a key player upstream of AKT, enhances the cell death induced by DNA-damaging agents. Building on these findings, clinical trials are underway to evaluate the effectiveness of combining PI3K inhibitors with DNA-based therapies in patients with TNBC [41,42].

It is clear that *PIK3CA* mutations are directly linked to TNBC subtypes, and the TNBC subtypes are correlated with the patient's epigenetics factors that contribute to significant clinical resistance. A recent study by Miquel et al. (2023) showed that younger African American women with tumors had distinct DNA methylation patterns compared to older African American women and white women of all ages. These epigenetic changes affected hormone and cell growth processes, which could contribute to more aggressive tumor characteristics in this patient group [43].

Moreover, having established the link between *PIK3CA* mutations and TNBC subtypes and the epigenetic profile, we now shift our focus to the specific molecular mechanisms by which *PIK3CA* orchestrates a tumor cell's propensity for tropism, including its impact on migration and invasion.

## The relationship between *PIK3CA* alterations and the acquisition of migratory and invasive phenotypes in TNBC cells

The interplay between *PIK3CA* alterations and the acquisition of migratory and invasive phenotypes in TNBC cells is a focal point in cancer research, offering insights into the underlying mechanisms of metastasis [44,45]. Numerous studies, including those by Hu et al. (2021) and Michal et al. (2017), have substantiated the correlation between *PIK3CA* mutations and heightened migratory and invasive capacities in TNBC [[23], [46], [47], [48]]. *PIK3CA* alterations, particularly gain-of-function mutations, amplify intracellular signaling cascades, fostering cytoskeletal dynamics and extracellular matrix (ECM) remodeling [49]. The vital mechanistic signaling pathway is the PI3K pathway which plays a key role in breast cancer. This pathway involves protein complexes called Class IA PI3Ks, which are made of two subunits: a regulatory subunit (P85α, P55α, P50α, P85β, P55γ) and a catalytic subunit (P110α*,* P110β, or P110δ) (Fig. 3) [50]. Signals from cell surface receptors normally activate these PI3Ks. As described earlier, the P110α subunit can directly activate PI3K signaling and promote cancer development. This adds to other known mechanisms like PTEN loss and AKT mutations, all of which contribute to uncontrolled cell growth through the PI3K/AKT pathway (See the Fig. 3) [51,52]. This intricate molecular orchestration empowers TNBC cells with enhanced migratory potential and invasive capabilities. Hu et al. (2021) in their investigation underscored this phenomenon, revealing a direct association between *PIK3CA* mutations and increased migration and invasion in TNBC cell types [23]. Furthermore, *PIK3CA*-induced activation of downstream effectors, notably matrix metalloproteinases (MMPs), emerges as a critical mediator of TNBC cell invasion. MMPs facilitate the degradation of the extracellular matrix, enabling cancer cells to breach tissue barriers and invade surrounding structures [53]. Comprehending the molecular intricacies of *PIK3CA*-driven migration and invasion holds profound implications for therapeutic strategies. Therefore, targeting *PIK3CA* alterations or downstream effectors involved in these processes may offer avenues for intervention to mitigate the aggressive behaviour of TNBC [54]. As research advances, unravelling the nuanced relationship between *PIK3CA* alterations and TNBC cell motility contributes not only to our understanding of metastasis but also holds promise for developing targeted therapies aimed at impeding the metastatic cascade in this challenging breast cancer subtype.

## The downstream signaling pathways activated by *PIK3CA* in TNBC cells for tumor tropism

Activating mutations in *PIK3CA* frequently lead to hyperactivation of the PI3K/AKT/mTOR pathway (See the Fig. 2), a key signaling axis implicated in diverse cellular processes [55]. Upon *PIK3CA* activation, PI3K phosphorylates PIP2 to generate PIP3. This event recruits AKT to the cellular membrane, where it undergoes phosphorylation and activation by PDK1 and the mTORC2. Activated AKT, in turn, phosphorylates numerous downstream effectors, including GSK-3, BAD, and FOXO transcription factors [56]. This phosphorylation acts in a negative way in the PI3K-PKB/AKT signaling pathway and shows its inhibitory effects on cancer cell growth and survival. However, FOXO transcription factors can further modulate the tumor progression and growth by maintaining cellular homeostasis which facilitates the metastasis and inducing therapy resistance in cells [57]. Furthermore, the PI3K/AKT/mTOR pathway intersects with various cellular processes influencing TNBC aggressiveness. Crosstalk with MAPK pathways, such as the Ras/Raf/MEK/ERK pathway (Fig. 2), accentuates the pro-proliferative effects of PI3K signaling. Additionally, *PIK3CA* mutations can impact EMT, fostering a more invasive and migratory TNBC phenotype [58].

## The PI3K pathway: a culprit in breast cancer's spread to bone

The PI3K pathway plays a critical role in many essential functions like cell growth, survival, movement, and attachment. Unfortunately, this same pathway also has a dark side - it contributes to the spread of breast cancer, particularly to bones.

Bone is a prime target for breast cancer metastasis, acting as a frequent first destination for cancer cells. This allows the cancer to establish a foothold and potentially spread further. Various studies have confirmed this, showing that bones and lungs are the most common sites where breast cancer relapses after initial treatment [[59], [60], [61]]. A study by Güth et al. (2014) involving over 1400 patients found that bone metastasis occurred in nearly 72% of patients with distant relapse, compared to 36% in the lungs, 20% in the liver, and a mere 6% in the brain [62].

## PI3K's partner in crime, the loss of PTEN

PTEN acts as a natural brake on the PI3K pathway, keeping its activity in check. In the case of bone metastasis, the loss of PTEN function emerges as a significant factor. Studies by Barbara et al. (2011) suggest that PTEN loss may be a predictor of poor prognosis for high-risk breast cancer patients with bone metastasis [63]. Interestingly, the way breast cancer spreads to different organs seems to involve distinct mechanisms. While the PI3K pathway is dominant in bone metastasis, lung metastasis appears to exploit a different strategy. Lung-derived bone morphogenetic proteins (BMPs) normally act as a barrier against metastasis. However, research suggests that cancer cells can manipulate these BMPs, rendering them ineffective. Another study by Song et al. (2016) identified a different signaling pathway, KRAS-PI3K-c-JUN, as a culprit in lung metastasis of breast cancer [64]. This highlights the complex and multifaceted nature of how breast cancer spreads to different organs.

## The crosstalk between PI3K/AKT/mTOR and other signaling pathways involved in tumor tropism

In the complex orchestration of cancer progression, the crosstalk between the PI3K/AKT/mTOR pathway and other signaling cascades emerges as a captivating saga, intricately weaving the narrative of tumor tropism [16]. This molecular interplay shapes the destiny of cancer cells, dictating their migratory prowess, invasive tendencies, and ability to colonize distant niches [65]. At the heart of this narrative is PI3K, the protagonist that instigates a cascade of events. As mentioned earlier, when activated, PI3K catalyzes the conversion of PIP2 to PIP3, beckoning AKT to the cellular membrane [66]. As AKT takes center stage, it phosphorylates key players in the ensemble, from GSK-3 to FOXO transcription factors, unleashing a symphony of pro-survival signals [[67], [68], [69]]. Enter the mTOR subplot, where this molecular conductor harmonizes with the PI3K/AKT melody, steering cellular processes toward growth and proliferation. However, the story turns riveting as the PI3K/AKT/mTOR narrative intersects with other signaling pathways in the tumor tropism saga. In the background, the Ras/Raf/MEK/ERK pathway emerges as a parallel storyline, complementing the PI3K/AKT/mTOR epic [70]. The crosstalk between these pathways amplifies the pro-proliferative effects, fuelling the cancer cell's relentless journey through the tangled thicket of tissues (Fig. 3).

As the tale unfolds, EMT emerges as a crucial subplot. Recently Glaviano et al. (2023) reviewed the PI3K/AKT/mTOR pathway and how it collaboratively works with TGF-β and Wnt signaling, shaping the cellular metamorphosis that propels cancer cells into a migratory and invasive state [71]. The angiogenesis storyline introduces VEGF, VEGFR-1, and VEGFR-2 as key supporting characters [72]. PI3K/AKT/mTOR intricately engages with these factors, influencing the creation of a vascular network that facilitates the cancer cell's journey by ensuring a nutrient-rich and oxygenated environment [70,71]. In this enthralling tale of molecular crosstalk, PI3K/AKT/mTOR stands as a central protagonist, weaving its narrative threads through multiple signaling pathways. The crosstalk orchestrates a symphony of signals, directing the intricate dance of tumor tropism with compelling precision. Yaqin et al. (2018) have provided insights into the therapeutic options for mutational TNBC [73]. Understanding these pathways is critical for developing targeted therapies to counteract the aggressive nature of TNBC and enhance the precision of therapeutic interventions.

This intricate signaling cascade orchestrated by *PIK3CA* has profound implications for tumor tropism. Enhanced cell survival, proliferation, migratory, and invasive capacities provide the foundation for TNBC cells to escape their primary site and establish distant metastases.

## *PIK3CA* on the modulation of chemokines, growth factors, and extracellular matrix interactions in the context of tumor tropism

*PIK3CA*, a linchpin in the PI3K/AKT/mTOR pathway, significantly influences tumor tropism by modulating chemokines, growth factors, and extracellular matrix (ECM) interactions. Various studies highlighted *PIK3CA*'s pivotal role in chemokine regulation and impacting immune cell recruitment [74,75]. In the realm of growth factors, *PIK3CA* mutations can alter responsiveness to stimuli, as seen in research highlighting its modulation of the EGFR pathway. Moreover, *PIK3CA* activation contributes to ECM alterations, influencing cancer cell invasion, as demonstrated by Wang et al. [76]. Understanding *PIK3CA*'s orchestration of these molecular processes offers insights into the complexities of tumor tropism, paving the way for targeted therapeutic strategies in aggressive cancers like triple-negative breast cancer [77]. Elucidating *PIK3CA*'s multifaceted influence on the tumor microenvironment and its role in tumor tropism has paved the way for targeted therapeutic strategies aimed at disrupting these pathways.

## Current therapeutic strategies targeting *PIK3CA* in TNBC

Current therapeutic strategies targeting *PIK3CA* in TNBC are at the forefront of precision medicine, aiming to disrupt the aberrant signaling cascade driving oncogenesis. Patients with breast cancer are particularly vulnerable to PI3K inhibition, and considerable progress has been made in targeting this pathway. Small-molecule inhibitors targeting PI3K, such as alpelisib, have shown promise in clinical trials [78], exhibiting efficacy in suppressing *PIK3CA*-mutant tumors [79]. Additionally, dual inhibitors like taselisib, which simultaneously target PI3K and the downstream effector AKT, have demonstrated encouraging results in preclinical studies. However, there are some limitations in the class of PI3K/AKT/mTOR dual-blockade agents regarding clinical activity due to adverse effects. For example, AZD2014, an mTOR catalytic inhibitor with preclinical evidence of higher activity, was found to be less effective in real-world scenarios than everolimus in HR-positive breast cancer [80]. More recently, multiple clinical studies have revealed that PI3K inhibitors are active in *PIK3CA* mutant HR^+^ breast cancer, including two studies with the pan-class I PI3K inhibitor buparlisib in BELLE-2 and BELLE-3 [[81], [82], [83]].

Combination therapies are also gaining traction, particularly in TNBC, where molecular heterogeneity poses challenges. In this scenario, mapping the molecular heterogeneity of patients adds advantages in targeting the *PIK3CA* gene and represent a step towards personalized treatment in breast cancer and TNBC.

Multi-protein inhibition, such as mTOR inhibition, AKT inhibition, and PI3K inhibition creates a feedback loop that may limit the activity of these agents. mTOR inhibition upregulates downstream RTKs, resulting in rebound AKT activation, while AKT inhibition induces FOXO-dependent transcription and RTK activation [81]. In parallel, PI3K inhibition not only prevents AKT activation but also results in increases MAPK signaling. These interactions illustrate the challenges of controlling and targeting specific pathways in cancer, which can lead to the development of resistance [84]. Combining PI3K inhibitors with standard chemotherapeutic agents or immune checkpoint inhibitors aims to enhance therapeutic outcomes synergistically. Notably, five FDA-approved PI3K inhibitors have been identified (copanlisib, idelalisib, umbralisib, duvelisib, and alpelisib). This has prompted clinicians and researchers to investigate additional PI3K inhibitors in preclinical and clinical studies, with the goal of finding potent inhibitors with high clinical efficacy, low toxicity, and optimal bioavailability [85].

Therefore, current therapeutic approaches primarily utilize small-molecule inhibitors targeting *PIK3CA*, such as alpelisib, or dual inhibitors like taselisib, which also block the downstream effector AKT. Clinical trials continue to investigate combination therapies pairing these PI3K inhibitors with standard chemotherapy or immunotherapy for enhanced efficacy. While challenges such as resistance mechanisms persist, the evolving landscape of *PIK3CA*-targeted therapies holds promise for improving clinical outcomes in TNBC. The dynamic nature of research and ongoing clinical investigations underscore the commitment to unraveling the intricacies of *PIK3CA*-driven TNBC and refining therapeutic strategies [78,86].

Despite these promising developments, targeting *PIK3CA* in TNBC remains a complex endeavor. Challenges such as tumor heterogeneity, limited understanding of predictive biomarkers, and acquired resistance mechanisms require innovative approaches and further research. However, these multifaceted challenges also fuel ongoing investigations and hold potential for significant breakthroughs in the fight against aggressive TNBC.

## Challenges and opportunities in developing targeted therapies based on *PIK3CA* status

Tumor heterogeneity is one of the most significant obstacles to *PIK3CA*-based therapy. Tumors display spatial and temporal variation in genetic alterations that make it difficult to precisely characterize *PIK3CA* mutations across regions. Another obstacle is identifying reliable biomarkers that can predict the response to *PIK3CA* inhibitors. Rigorous biomarkers are critical for patient selectivity and treatment effectiveness. Integrating various data sets requires advanced bioinformatics tools that can sift through large molecular datasets and identify relevant biomarkers amid the noise. Proteomic profiling has the potential to reveal the signaling pathways that underlie resistance mechanisms. While *PIK3CA* inhibitors may initially be promising, acquired resistance can limit their long-term effectiveness. It is difficult to interpret complex data to find actionable targets. These hurdles are the main challenges in developing targeted therapies based on *PIK3CA* status. Comprehensive genomic profiling, such as next-generation sequencing, allows for a detailed understanding of *PIK3CA* mutations, aiding in patient stratification for tailored treatments [87].

In most cases, frequent alteration in the mutation of *PIK3CA* is a real challenge to target for BC therapy [88]. Boyault et al. (2012) performed a study on 120 human primary breast tumors and found that *PIK3CA* is one of the five genes commonly mutated in breast cancer, with a substantial proportion of tumors carrying mutations in either *TP53* or in genes of the PI3K pathway (*PIK3CA, PTEN*, or *AKT1*) [89]. Emerging evidence supports the efficacy of PI3K inhibitors, like alpelisib and taselisib, in *PIK3CA*-mutant cancers, showing improved outcomes in clinical trials [85]. Combination therapies that incorporate PI3K inhibitors with other targeted agents or immunotherapies offer a strategic approach. Clinical trials, such as NCT03742102, NCT04191499, and NCT04975958, explore these combinations, demonstrating a shift toward synergistic treatment strategies [[90], [91], [92]]. An interesting study by Jiang et al. (2014) reported that neoadjuvant chemotherapy also reduces *PIK3C*A mutations, supporting its use in combination with chemotherapy [93]. While challenges persist, ongoing research underscores the potential of personalized therapies based on *PIK3CA* status. Kalinsky et al. (2009) showed that patients with mutated tumors (192 tumors: 32.5%) have improved progression-free, distant progression-free, overall, and breast cancer-specific survival. [94]. The dynamic landscape of clinical trials and evolving treatment paradigms highlight the commitment to refining strategies that may reshape the future of cancer care.

## Conclusions

Despite the challenges that remain, the rapid evolution of *PIK3CA*-targeted therapies, including combination approaches and the exploration of novel targets, offers significant promise in revolutionizing the treatment of breast cancers with *PIK3CA* alterations. Recent clinical trials established that small molecules like Alpelisib [78], and copanlisib [95] are effective in targeting the mutated *PIK3CA* gene and its pathway. Consequently, various molecules and downstream pathways, such as AKT-mTOR pathways, are now the primary focus for clinical treatment. Moreover, we can target various chemokines and growth factors that are parallay involved in the TNBC tropism (Table-2). Another unexplored area is mapping the tumor microenvironment in the context of *PIK3CA* gene mutations, which could provide greater accuracy in developing clinical strategies. . The clinical progress reflects the dedication of researchers and clinicians committed to deciphering the complexities of aggressive breast cancers like TNBC. While hurdles such as tumor heterogeneity and resistance mechanisms remain, ongoing research and clinical trials hold the key to overcoming these obstacles. These efforts bring hope to patients, with the potential to significantly improve outcomes and tailor treatment strategies to combat even the most challenging breast cancers.

**Table 2:** This table summarizes the impacts of chemokines and growth factors on tumor tropism via interactions with the *PIK3CA* gene.

Chemokines	Interaction with *PIK3CA*	Role in Tumor Tropism	Refs.
CXCL12 (SDF-1)	*PIK3CA* amplifies CXCL12 signaling and enhances recruitment of CXCR4-positive cells	Attract breast cancer cells to favorable areas for growth and guide their migration.	[74,96]
CCL2 (MCP-1)	*PIK3CA* modulates CCL2-induced chemotaxis which influences breast cancer tumor microenvironment.	Facilitates recruitment of monocytes and macrophages leading to tumor-promoting interactions and invasive behavior.	[97]
CXCL8 (IL-8)	*PIK3CA* activates CXCL8 expression which promotes angiogenesis through CXCL8/IL-8 signaling.	Induces migration and invasion of breast cancer cells which contributes to tumor progression and metastasis.	[98]
CXCL1 and CXCL2	*PIK3CA* enhances CXCL1 and CXCL2 expression and impacts the recruitment of neutrophils to the tumor microenvironment.	This factor shapes the premetastatic niche and fuels metastasis by enabling breast cancer cell movement and invasion.	
CCL5 (RANTES)	*PIK3CA* modulates CCL5-induced chemotaxis by influencing immune cell recruitment.	Regulates migration of breast cancer cells which contributes to the establishment of a pro-tumorigenic environment.	[99]

Growth factors	Interaction with *PIK3CA*	Role in Tumor Tropism	Refs.
EGF	*PIK3CA* activates downstream signaling pathways in response to EGF stimulation. Crosstalk between PI3K/AKT/mTOR and EGF pathways influences migration and invasion.	Fuel the breast cancer progression by promoting cell proliferation, survival, migration, and invasion.	[100]
IGF-1	*PIK3CA* signaling integrates with IGF-1 receptor activation. Interaction enhances the migration and invasion potential of breast cancer cells.	Stimulates cell proliferation and survival, promoting tumor growth which contributes to the acquisition of invasive phenotypes.	[101,102]
FGF	Mutated *PIK3CA* modulates FGF-induced signaling cascades. Interplay with FGF pathways influences tumor cell migration and invasion.	Stimulates new blood vessel growth and creates a metastasis-friendly environment, this process fuels tumor progression	[103]
HGF	*PIK3CA* interacts with the HGF/MET signaling pathway. Cooperation with HGF promotes metastasis through EMT (Epithelial-Mesenchymal Transition).	Enhances cell motility and invasiveness of breast cancer cells by facilitating the transition of cancer cells to a more vascular network within the tumor.	[104]
PDGF	*PIK3CA* participates in PDGF-induced signaling pathways. Interaction influences tumor cell migration and invasion.	Contributes to angiogenesis, fostering the development of a vascular network within the tumor.	[104]
VEGF	*PIK3CA* contributes to VEGF-induced angiogenesis. Influences endothelial cell migration and vascular permeability.	Promotes the formation of new blood vessels, facilitating tumors which supports the establishment of a vascular network for metastatic potential.	[[105], [106], [107]]
TGF-β	*PIK3CA* regulates downstream signaling in response to TGF-β stimulation. Interaction contributes to enhanced invasion and metastasis.	Modulates the epithelial-mesenchymal transition (EMT) and impacts the acquisition of mesenchymal phenotypes in cancer cells.	[108]
NGF	*PIK3CA* is implicated in NGF-induced signaling pathways. The interaction may contribute to neural invasion and metastasis.	Influences neuronal support for cancer cells and their invasive behaviour. Impacts the tropism of breast cancer cells towards neural tissues.	
GH	*PIK3CA* participates in GH-mediated intracellular signaling. The interaction may influence the migratory and invasive potential.	Enhances cell growth and survival, and leads to tumor progression. Contributes to the tropism of breast cancer cells towards specific sites.	[109]
IGFBP	*PIK3CA* may influence IGFBP-3 regulation. The interaction may have downstream effects on cell proliferation and survival	Impacts the bioavailability and activity of IGF-1, affecting tumor tropism. Influences the growth-promoting environment within the tumor and impacts the bioavailability and the activity of IGF-1, affecting the tumor tropism.	[102]

## Funding

The authors did not receive any funds for this work.

## CRediT authorship contribution statement

**Sumit Mallick:** Writing – review & editing, Writing – original draft, Conceptualization. **Asim K Duttaroy:** Writing – review & editing. **Suman Dutta:** Writing – review & editing.

## Declaration of competing interest

The Authors declare no conflict of interest.
